# Scaling of Brain Metabolism and Blood Flow in Relation to Capillary and Neural Scaling

**DOI:** 10.1371/journal.pone.0026709

**Published:** 2011-10-28

**Authors:** Jan Karbowski

**Affiliations:** Institute of Biocybernetics and Biomedical Engineering, Polish Academy of Sciences, Warsaw, Poland; University of Pittsburgh, United States of America

## Abstract

Brain is one of the most energy demanding organs in mammals, and its total metabolic rate scales with brain volume raised to a power of around 5/6. This value is significantly higher than the more common exponent 3/4 relating whole body resting metabolism with body mass and several other physiological variables in animals and plants. This article investigates the reasons for brain allometric distinction on a level of its microvessels. Based on collected empirical data it is found that regional cerebral blood flow CBF across gray matter scales with cortical volume 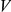 as 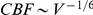, brain capillary diameter increases as 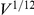, and density of capillary length decreases as 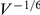. It is predicted that velocity of capillary blood is almost invariant (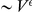), capillary transit time scales as 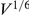, capillary length increases as 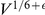, and capillary number as 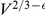, where 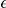 is typically a small correction for medium and large brains, due to blood viscosity dependence on capillary radius. It is shown that the amount of capillary length and blood flow per cortical neuron are essentially conserved across mammals. These results indicate that geometry and dynamics of global neuro-vascular coupling have a proportionate character. Moreover, cerebral metabolic, hemodynamic, and microvascular variables scale with allometric exponents that are simple multiples of 1/6, rather than 1/4, which suggests that brain metabolism is more similar to the metabolism of aerobic than resting body. Relation of these findings to brain functional imaging studies involving the link between cerebral metabolism and blood flow is also discussed.

## Introduction

It is well established empirically that whole body metabolism of resting mammals scales with body volume (or mass) with an exponent close to 3/4, which is known as Kleiber's law [1], [2], [3], [4]. The same exponent or its simple derivatives govern the scalings of respiratory and cardiovascular systems in mammals and some other physiological parameters in animals and plants [2], [3], [5]. Because of its almost ubiquitous presence, the quarter power has often been described as a general law governing metabolism and blood circulation, and several formal models explaining its origin have been proposed that still cause controversy [6], [7], [8], [9]. However, as was found by the author [10], the brain metabolism at rest seems to follow another scaling rule. Total brain metabolic rate (both oxygen and glucose) scales with brain volume with an exponent 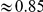, or close to 5/6 [10]. Consequently, the volume-specific cerebral metabolism decreases with brain size with an exponent around 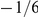, and this value is highly homogeneous across many structures of gray matter [10]. The origin of these cerebral exponents has never been explained, although it is interesting why brain metabolism scales different than metabolism of other systems.

The brain, similar to other organs, uses capillaries for delivery of metabolic nutrients (oxygen, glucose, etc.) to its cells [11]. Moreover, numerical density of cerebral capillaries is strongly correlated with brain hemodynamics and metabolism [12], [13]. However, the cerebral microvascular network differs from other non-cerebral networks in two important ways. First, in the brain there exists a unique physical border, called the brain-blood barrier, which severely restricts influx of undesired molecules and ions to the brain tissue. Second, cerebral capillaries exhibit a large degree of physical plasticity, manifested in easy adaptation to abnormal physiological conditions. For instance, during ischemia (insufficient amount of oxygen in the brain) capillaries can substantially modify their diameter to increase blood flow and hence oxygen influx [14], [15], [16]. These two factors, i.e. structural differences and plasticity of microvessels, can in principle modify brain metabolism in such a way to yield different scaling rules in comparison to e.g. lungs or muscles. Another, related factor that may account for the uncommon brain metabolic scaling is the fact that brain is one of the most energy expensive organs in the body [10], [17]. This is usually attributed to the neurons with their extended axons and dendrites, which utilize relatively large amounts of glucose and ATP for synaptic communication [18], [19].

The main purpose of this paper is to determine scaling laws for blood flow and geometry of capillaries in the brain of mammals. Are they different from those found or predicted for cardiovascular and respiratory systems? If so, do these differences account for brain metabolic allometry? How the scalings of blood flow and capillary dimensions relate to the scalings of neural characteristics, such as neural density and axon (or dendrite) length? This study might have implications for expanding of our understanding of mammalian brain evolution, in particular the relationship between brain wiring, metabolism, and its underlying microvasculature [10], [20], [21]. The results can also be relevant for research involving the microvascular basis of brain functional imaging studies, which use relationships between blood flow and metabolism to decipher regional neural activities [22], [23].

## Results

The data for brain circulatory system were collected from different sources (see Materials and Methods). They cover several mammals spanning 3–4 orders of magnitude in brain volume, from mouse to human.

### 1. Empirical scaling data

**Figure 1:**
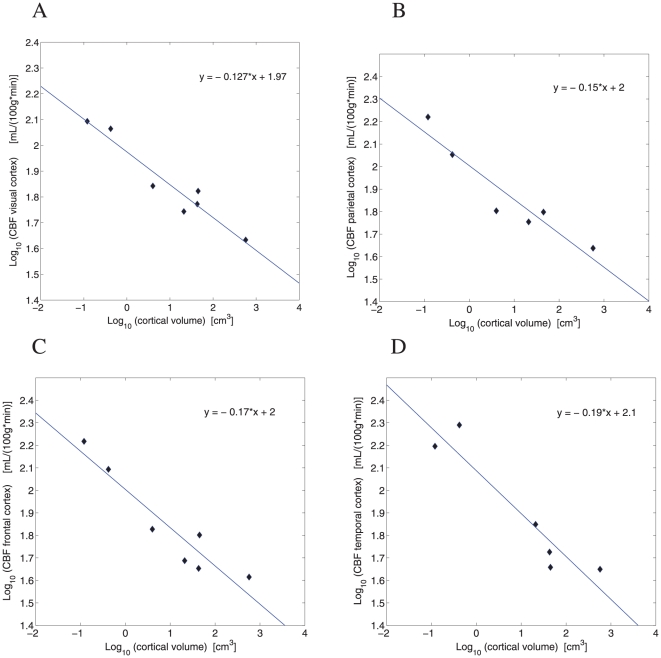
Scaling of cerebral blood flow CBF in the cortical gray matter. (A) Visual cortex: 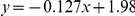 (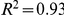, 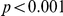). 95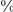 confidence interval for the slope CI = (−0.168,−0.086). (B) Parietal cortex: 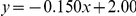 (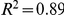, 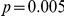), slope CI = (−0.222,−0.078). (C) Frontal cortex: 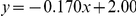 (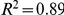, 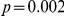), slope CI = (−0.239,−0.100). (D) Temporal cortex: 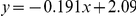 (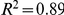, 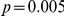), slope CI = (−0.286,−0.096).

**Figure 2:**
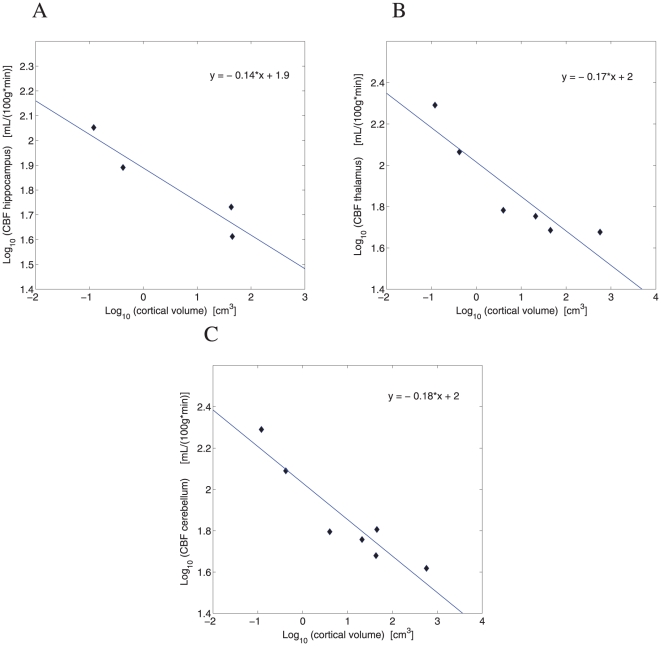
Scaling of cerebral blood flow CBF in the subcortical gray matter. (A) Hippocampus: 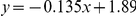 (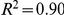, 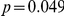), slope CI = (−0.271,0.000). (B) Thalamus: 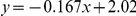 (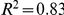, 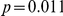), slope CI = (−0.272,−0.062). (C) Cerebellum: 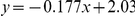 (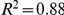, 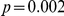), slope CI = (−0.252,−0.102).

Cerebral blood flow CBF in different parts of mammalian gray matter decreases systematically with gray matter volume, both in the cortical and subcortical regions (Fig. 1). In the cerebral cortex, the scaling exponent for regional CBF varies from 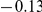 for the visual cortex (Fig. 1A), 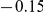 for the parietal cortex (Fig. 1B), 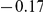 for the frontal cortex (Fig. 1C), to 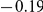 for the temporal cortex (Fig. 1D). The average cortical exponent is 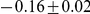. In the subcortical regions, the CBF scaling exponent is 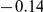 for hippocampus (Fig. 2A), 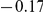 for thalamus (Fig. 2B), and 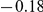 for cerebellum (Fig. 2C). The average subcortical exponent is identical with the cortical one, i.e., 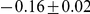, and both of them are close to 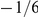. It is interesting to note that almost all of the cortical areas (except temporal cortex) have scaling exponents whose 95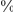 confidence intervals do not include a quarter power exponent 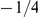.

**Figure 3:**
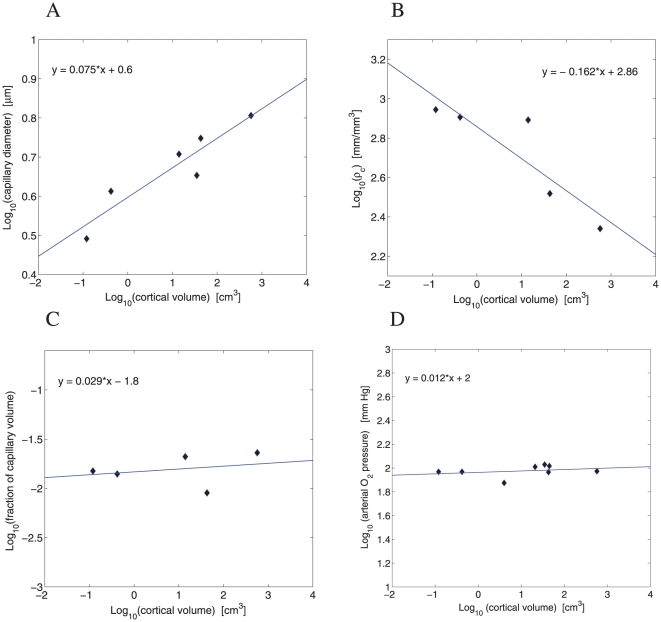
Scaling of brain capillary characteristics against brain size. (A) Capillary diameter scales against cortical gray matter volume with the exponent 0.075 (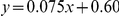, 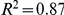, 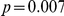), exponent CI = (0.034,0.117). (B) Volume density of capillary length 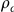 scales with the exponent 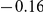 (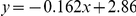, 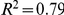, 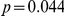), exponent CI = (−0.316,−0.008). (C) Fraction of capillary volume 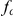 in gray matter is essentially independent of brain size (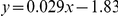, 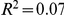, 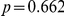), the same as (D) the arterial partial oxygen pressure (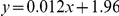, 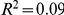, 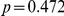).

The microvessel system delivering energy to the brain consists of capillaries. The capillary diameter increases very weakly but significantly with brain size, with an exponent of 0.08 (Fig. 3A). On the contrary, the volume-density of capillary length decreases with brain size raised to a power of 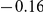 (Fig. 3B). Thus, the cerebral capillary network becomes sparser as brain size increases. Despite this, the fraction of gray matter volume taken by capillaries is approximately independent of brain size (Fig. 3C). Another vascular characteristic, the arterial partial oxygen pressure, is also roughly invariant with respect to brain volume (Fig. 3D).

**Figure 4:**
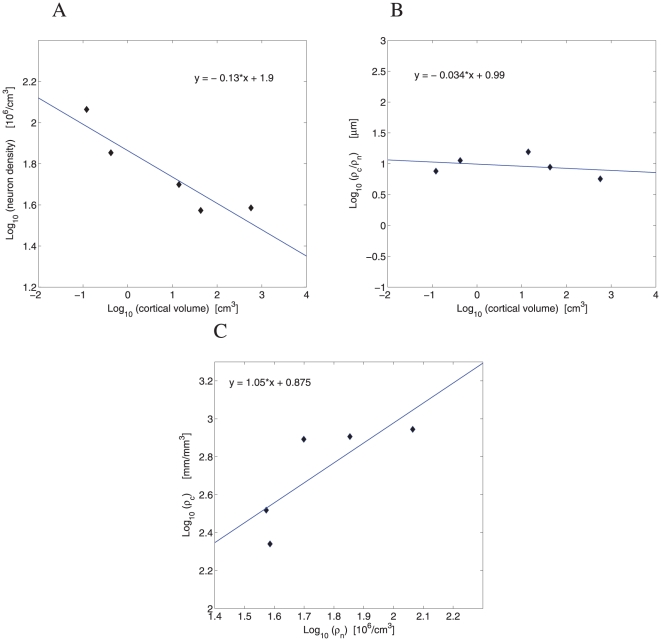
Neuron density versus capillary length density in the cerebral cortex. (A) Across our sample of mammals, the cortical neuron number density 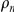 scales against cortical volume with the exponent 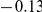 (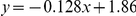, 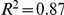, 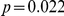), exponent CI = (−0.221,−0.036). (B) The ratio of the density of capillary length 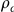 to the density of neurons 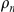 in the cortex does not correlate with brain size (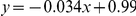, 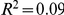, 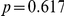), exponent CI = (−0.228,0.161). (C) The log-log dependence of the capillary length density 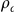 on neuron density 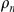 gives the exponent of 1.05 (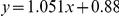, 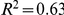, 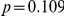).

A degree of neurovascular coupling can be characterized by geometric relationships between densities of capillaries and neurons. Scaling of the density of neuron number in the cortical gray matter is not uniform across mammals [24], [25], [26]. In fact, the scaling exponent depends to some extent on mammalian order and the animal sample used [26]. For the sample of mammals used in this study, it is found that cortical neuron density decreases with cortical gray matter volume with an exponent of 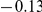 (Fig. 4A). This exponent is close to the exponent for the scaling of capillary length density, which is 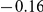 (Fig. 3B). Consistent with that, the ratio of cortical capillary length density to neuron density across mammals is approximately constant and independent of brain size (Fig. 4B). Typically, there is about 10 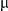
m of capillaries per cortical neuron. The scaling dependence between the two densities yields an exponent close to unity (Fig. 4C), which shows a proportionality relation between them.

**Figure 5:**
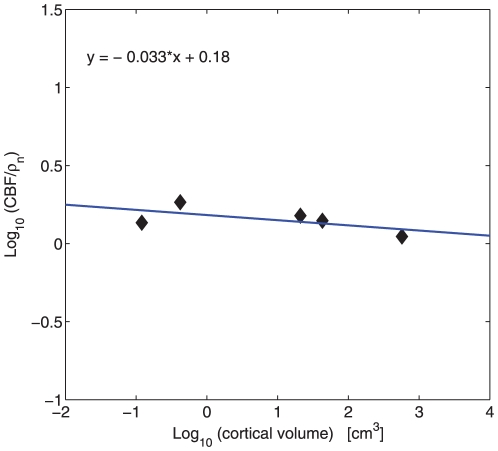
Invariance of cerebral blood flow per cortical neuron across mammals. The ratio of CBF to neuron density 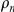 in the cerebral cortex does not correlate significantly with brain size (log-log plot yields 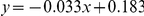, 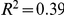, 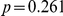). The value of CBF for each species is the arithmetic mean of regional CBF across cerebral cortex.

Cerebral blood flow CBF scales with brain volume the same way as does capillary length density (Figs. 1,2,3B), and thus, CBF should also be related to neural density. Indeed, in the cerebral cortex the ratio of the average CBF to cortical neural density is independent of brain scale (Fig. 5). This means that the average amount of cortical blood flow per neuron is invariant among mammals, and about 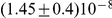 mL/min. Taken together, the findings in Figs. 4 and 5 suggest a tight global correlation between neurons and their energy supporting microvascular network.

### 2. Theoretical scaling rules for cerebral capillaries

Below I derive theoretical predictions for the allometry of brain capillary characteristics, such as: capillary length and radius, capillary number, blood velocity, and time taken by blood to travel through a capillary. I also find relationships connecting cerebral metabolic rate and blood flow with neuron density. The following assumptions are made in the analysis: (i) Oxygen consumption rate in gray matter CMR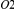 scales with cortical gray matter volume 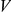 as 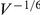, in accordance with Ref. [10]; (ii) Capillary volume fraction, 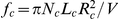, is invariant with respect to 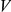, which follows from the empirical results in Fig. 3C. The symbol 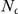 denotes total capillary number in the gray matter, 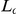 is the length of a single capillary segment, and 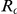 is its radius; (iii) Driving blood pressure 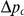 through capillaries is independent of brain size, which is consistent with a known fact that arterial blood pressure (both systolic and diastolic) of resting mammals is independent of body size [27], [28], [29]; (iv) Partial oxygen pressure 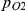 in capillaries is also invariant, which is consistent with the empirical data in Fig. 3D on the invariance of arterial oxygen pressure; (v) Cerebral blood flow CBF is proportional to oxygen consumption rate CMR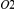, due to adaptation of capillary diameters to oxygen demand.

The cerebral metabolic rate of oxygen consumption CMR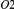, according to the modified Krogh model [11], [14], is proportional to the product of oxygen flux through capillary wall and the tissue-capillary gradient of oxygen pressure 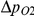, i.e.
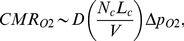
(1)where 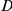 is the oxygen diffusion constant in the brain. The dependence of CMR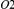 on capillary radius in this model has mainly a logarithmic character, and hence it is neglected as weak. Since oxygen pressure in the brain tissue is very low [30], the pressure gradient 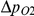 is essentially equal to the capillary oxygen pressure 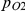. Consequently, the formula for CMR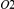 simplifies to 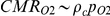, where 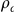 is the density of capillary length 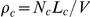.

From the assumptions (i) and (iv) we obtain that capillary length density 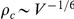. Additionally, from (ii) we have 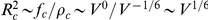, implying that capillary radius (or diameter) 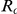 scales as 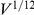. Consequently capillary diameter does not increase much with brain magnitude. As an example, a predicted capillary diameter for elephant with its cortical gray matter volume 1379 cm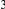 [31] is 7.2 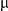
m, which does not differ much from those of rat (4.1 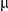
m [15], [32]) or human (6.4 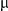
m [33], [34]), who have corresponding volumes 3450 and 2.4 times smaller.

The blood flow 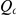 through a capillary is governed by a modified Poiseuille's law in which blood viscosity depends on capillary radius [35]:
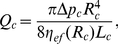
(2)where 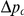 is the axial driving blood pressure along a capillary of length 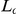, and 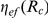 is the capillary radius dependent effective blood viscosity. The latter dependence has a nonmonotonic character, i.e. for small diameters the viscosity 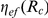 initially decreases with increasing 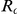, reaching a minimum at diameters about 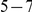 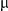
m. For 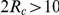 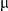
m the blood viscosity 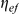 slowly increases with 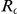 approaching its bulk value for diameters 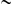 500 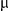
m. This phenomenon is known as the Fahraeus-Lindqvist effect [36]. In general, blood viscosity in narrow microvessels depends on microvessel thickness because red blood cells tend to deform and place near the center of capillary leaving a cell-free layer near the wall [35], [37]. These two regions have significantly different viscosities, with the cell-free layer having essentially plasma viscosity 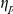, which is much smaller than the bulk (or center region) viscosity 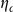. The formula relating the effective blood viscosity 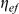 with capillary radius and both viscosities 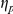 and 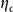 is given by [35]:
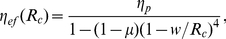
(3)where 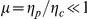, and 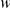 is the thickness of cell-free layer.

For capillary radiuses relevant for the brain, i.e. 1.5 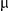
m
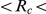
3.5 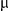
m (see Suppl. Table S2), the ratio 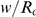 increases with increasing 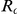, which causes a decline in the effective blood viscosity down to its minimal value at 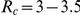 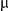
m (Table 1). Using the data in Table 1 taken from [35], we can approximate the denominator in Eq. (3) for this range of radiuses by a simple, explicit function of 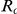. The best fit is achieved with a logarithmic function, i.e. 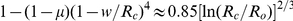, where 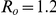 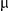
m (Table 1). As a result, the effective blood viscosity takes a simple form:
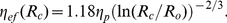
(4)


**Table 1:** Parameters affecting the effective blood viscosity.

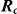 [ 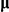 m]	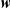 [ 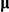 m]	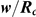	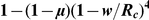	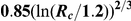
1.5	0.07	0.05	0.27	0.31
2.0	0.30	0.15	0.54	0.54
2.5	0.60	0.24	0.71	0.69
3.0	0.90	0.30	0.79	0.80

Data for 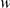 and 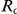 were collected from [35]. The value of 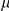 was taken as 1/8. The last column represents values of the fitting function to the function in the fourth column.

Cerebral blood flow CBF in the brain gray matter is defined as 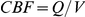, where 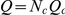 is the total capillary blood flow through all 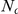 capillaries. Thus CBF is given by
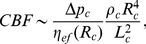
(5)or
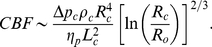
(6)


We can rewrite the logarithm present in Eq. (6), in an equivalent form, as a power function 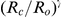 with a variable exponent 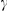 given by (see Appendix S1 in the Supp. Infor.):
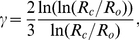
(7)so that CBF becomes
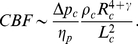
(8)


The exponent 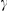 in this equation can be viewed as a correction due to non-constant blood viscosity (Fahraeus-Lindqvist effect [36]). The dependence of 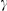 on the capillary diameter is shown in Table 2. Because in general 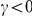, its presence in Eq. (8) reduces the power of 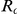. However, this effect is weak for medium and large brains as 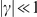. Even for a small rat brain the relative influence of 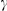 is rather weak, since 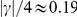. In contrast, for very small brains, such as mouse, the effect caused by 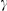 is strong (Table 2), which reflects a sharp increase in the effective blood viscosity for the smallest capillaries [35], [37].

**Table 2:** Exponent 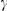 as a function of capillary diameter.

Species	mouse	rat	cat	dog	monkey	human
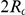 [ 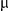 m]	3.1	4.1	5.1	4.5	5.6	6.4
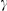	−3.51	−0.77	−0.25	−0.49	−0.13	−0.01

Data for 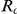 were taken from Suppl. Inform. Table S2 (references therein).

Now we are in a position to derive scaling rules for the capillary length segment 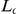, capillary blood velocity 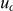, and the number of capillaries 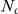. From Eq. (8), using the assumptions (i), (iii), and (v), we obtain 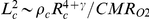, which implies that 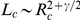 (viscosity of blood plasma is presumably independent of brain scale [38]). Consequently, 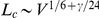, i.e. capillary length should weakly increase with brain size. Although there are no reliable data on 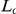, we can compare our prediction with the measured intercapillary distances, which generally should be positively correlated with 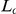. Indeed, the mean intercapillary distance in gray matter increases with increasing brain volume, and is 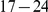 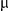
m in rat [39], 24 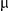
m in cat [40], and 58 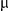
m in human [33].

Average velocity 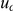 of blood flow in brain capillaries is given by 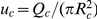. Using the expressions for 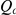 and 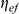, we get 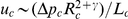. Since 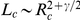 above, and using the assumption (iii), we obtain 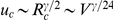. Thus, capillary blood velocity is almost independent of brain size for medium and large brains, as then 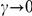 (Table 2). For very small brains, instead, there might be a weak dependence. A related quantity, the blood transit time 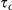 through a capillary, defined as 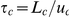, scales as 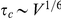, regardless of the brain magnitude. This indicates that 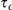 and CBF are inversely related across different species, 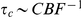, because of their scaling properties.

We can find the scaling relation for the total number of capillaries 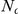 from the volume-density of capillary length 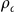. We obtain 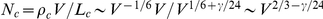, i.e. the exponent for 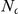 is close to 2/3 for not too small brains. As an example, the number of capillary segments in the human cortical gray matter should be 123 times greater than that in the rat (cortical volumes of both hemispheres in rat and human are 0.42 cm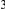 [24] and 572.0 cm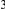 [41], respectively).

As was shown above, CMR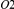 must be proportional to the volume density of capillary length 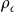 (Eq. 1). On the other hand, the empirical results in Fig. 4 indicate that 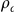 is roughly proportional to neuron density 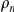. Thus, we have approximately CMR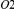 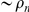 across different mammals. This implies that oxygen metabolic energy per neuron in the gray matter should be approximately independent of brain size. Exactly the same conclusion was reached before in a study by Herculano-Houzel [26], based on independent data analysis. Moreover, since cortical CMR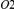 and CBF scale the same way against brain size, we also have 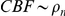, which is confirmed by the results in Fig. 5. In other words, both cerebral metabolic rate and blood flow per neuron are scale invariant.

## Discussion

### 1. General discussion

The summary of the scaling results is presented in Table 3. Some of these allometric relations are directly derived from the experimental data (CBF, 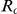, 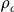, 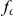, 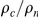, CBF/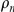), and others are theoretically deduced (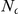, 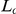, 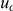, 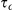). The interesting result is that cerebral blood flow CBF in gray matter scales with cortical gray matter volume raised to a power of 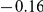. The similar exponent governs the allometry of cortical metabolic rate CMR [10], which indicates that brain metabolism and blood flow are roughly linearly proportional across different mammals. This conclusion is compatible with several published studies that have shown the proportionality of CMR and CBF on a level of a single animal (rat, human) across different brain regions [12], [42].

**Table 3:** Summary of scalings for brain capillaries and hemodynamics against cortical gray matter volume 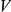.

Parameter	Scaling rule
Capillary radius, 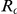	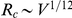
Capillary length density, 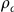	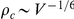
Capillary volume fraction, 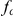	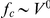
Total capillary number, 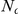	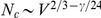
Capillary segment length, 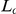	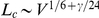
Capillary blood velocity, 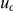	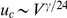
Capillary transit time, 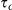	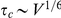
Capillary oxygen pressure, 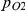	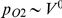
Capillary length per neuron, 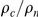	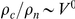
Cerebral blood flow, 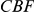	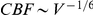
Blood flow per neuron, 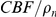	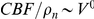
Oxygen consumption rate, 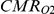	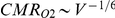
Oxygen use per neuron, 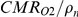	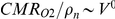

The coupling between CMR and CBF manifests itself also in their relation to the number of neurons. In this respect, the present study extends the recent result of Herculano-Houzel [26] about the constancy of metabolic energy per neuron in the brains of mammals, by showing that also cerebral blood flow and capillary length per neuron are essentially conserved across species. There are approximately 10 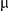
m of capillaries and 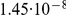 mL/min of blood flow per cortical neuron (Figs. 4 and 5; Supp. Tables S2 and S3). This finding suggests that not only brain metabolism but also its hemodynamics and microvascularization are evolutionarily constrained by the number of neurons. This mutual coupling might be a result of optimization in the design of cerebral energy expenditure and blood circulation.

It should be underlined that both CBF and CMR scale with brain volume with the exponent about 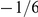, which is significantly different from the exponent 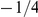 relating whole body resting specific metabolism with body volume [1], [2], [3]. Instead, the cerebral exponent 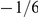 is closer to an exponent 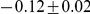 characterizing maximal body specific metabolic rate and specific cardiac output in strenuous exercise [43], [44]. In this sense, the brain metabolism and its hemodynamics resemble more the metabolism and circulation of exercised muscles than other resting organs, which is in line with the empirical evidence that brain is an energy expensive organ [10], [17], [18]. This may also suggest that there exists a common plan for the design of microcirculatory system in different parts of the mammalian body that uses the same optimization principles [45].

The results of this study show that as brain increases in size its capillary network becomes less dense, i.e. the densities of both capillary number and length decrease, respectively as 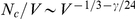 and 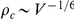 (Table 3). Contrary to that, the capillary dimensions increase weakly with brain volume, their radius as 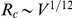 and their length segment as 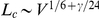, which are sufficient to make the fraction 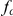 of capillary volume in the gray matter to be scale invariant (Table 3). The correction 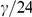 appearing in the scaling exponents for 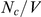 and 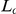 reflects the fact that blood viscosity depends on capillary radius (Fahraeus-Lindqvist effect [36]). This correction is however small for sufficiently large brains, generally for brains larger or equal to that of rat, for which typical values of 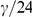 are in the range from 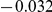 to 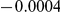 (Table 2). On the contrary, for brains of mouse size or smaller, this correction is substantial, about 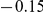, which implies that for very small brains 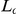 is essentially constant.

Despite the changes in the geometry of microvessels, the velocity of capillary blood 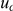 is almost scale invariant for not too small brains (exponent 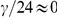; Table 3). This prediction agrees with direct measurements of velocity in the brains of mouse, rat, and cat, which does not seem to change much, i.e. it is in the range 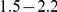 mm/sec [40], [46]. Consequently the transit time 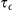 through a capillary increases with brain size as 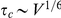, i.e. the scaling exponent is again 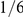. Another variable that seems to be independent of brain scale is partial oxygen pressure in cerebral capillaries (Table 3), which is consistent with the empirical findings in Fig. 3D on the invariance of oxygen pressure in arteries, as the two circulatory systems are mutually interconnected.

### 2. Capillary scaling in cerebral and non-cerebral tissue

The above scaling results for the brain can be compared with available analogous scaling rules for pulmonary, cardiovascular, and muscle systems. For these systems, it was proposed (no direct measurements) that partial oxygen pressure in capillaries should decline weakly with whole body volume (or organ volume as lung and heart volumes, 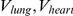, scale isometrically with body volume [2]) with an exponent around 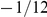, to account for the whole body specific metabolic exponent 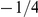 [47], [48]. In the resting pulmonary system, the capillary radius as well as the density of capillary length scale the same way as they do in the brain, i.e., with the exponents 1/12 and 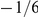, respectively, against system's volume [49]. Also, the capillary blood velocity in cerebral and non-cerebral tissues scale similarly, at least for not too small volumes, i.e. both are scale invariant [2], [3] (Table 3). However, the number of capillaries and capillary length seem to scale slightly different in the resting lungs, i.e. 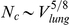 and 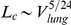 [47], although the difference can be very mild. For the resting heart, it was predicted (again, no direct measurements) that 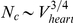, and blood transit time through a capillary 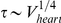 [48], i.e. the exponents are multiples of a quarter power and are slightly larger than those for the brain (Table 3). Interestingly, for muscles and lungs in mammals exercising at their aerobic maxima, the blood transit time scales against body mass with an exponent close to 1/6 [50], which is the same as in the brain (Table 3). This again suggests that brain metabolism is similar to the metabolism of other maximally exercised organs. Overall, the small differences in the capillary characteristics among cerebral and non-cerebral resting tissues might account for the observed differences in the allometries of brain metabolism and whole body resting metabolism. In particular, the prevailing exponent 1/6 found in this study for brain capillaries, instead of 1/4, seems to be a direct cause for the distinctive brain metabolic scaling.

### 3. Brain microvascular network vs. neural network

The interesting question from an evolutionary perspective is how the allometric scalings for brain capillary dimensions relate to the allometry of neural characteristics. The neural density 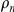 (number of cortical neurons 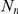 per cortical gray matter volume 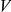) scales with cortical volume with a similar exponent as does the density of capillary length 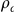 (Fig. 4A). Thus, as a coarse-grained global description we have approximately 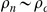 (Fig. 4B,C), or 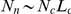. The latter relation means that the total number of neurons is roughly proportional to the total length of capillaries, or equivalently, that capillary length per cortical neuron is conserved across different mammals. This cross-species conclusion is also in agreement with the experimental data for a single species. In particular, for mouse cerebral cortex it was found that densities of neural number and microvessel length are correlated globally across cortical areas (but not locally within a single column) [51]. Moreover, since axons and dendrites occupy a constant fraction of cortical gray matter volume (roughly 1/3 each; [52], [53]), we have 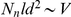, where 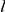 and 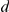 are respectively axon (or dendrite) length per neuron and diameter. Furthermore, because the average axon diameter 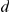 (unmyelinated) in the cortical gray matter is approximately invariant against the change of brain scale [52], [54], we obtain the following chain of proportionalities: 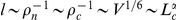, where the exponent 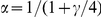. For medium and large brains, 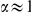, implying a nearly proportional dependence of axonal and dendritic lengths on capillary segment length. For very small brains (roughly below the volume of rat brain), 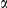 can be substantially greater than 1, suggesting a non-linear dependence between capillary and neural sizes.

Given that the main exchange of oxygen between blood and brain takes place in the capillaries, these results suggest that metabolic needs of larger brains with greater but numerically sparser neurons must be matched by appropriately longer yet sparser capillaries. This finding reflects a rough, global relationship, which might or might not be related to the fact that during development neural and microvessel wirings share mutual mechanisms [20], [55]. At the cortical microscale, however, things could be more complicated, and a neuro-vascular correlation might be weaker, as both systems are highly plastic even in the adult brain (e.g. [56]). Regardless of its nature and precise dependence, the neuro-vascular coupling might be important for optimization of neural wiring [53], [57], [58]. In fact, neural connectivity in the cerebral cortex is very low, and it decreases with brain size [58], [59], similar to the density of capillary length (Fig. 3B, Table 3). To make the neural connectivity denser, it would require longer axons and consequently longer capillaries. That may in turn increase excessively brain volume and its energy consumption, i.e. the costs of brain maintenance. As a result, the metabolic cost of having more neural connections and synapses for storing memories might outweigh its functional benefit.

The brain metabolism is obviously strictly related to neural activities. In general, higher neural firing rates imply more cerebral energy consumed [18], [19]. It was estimated, based on a theoretical formula relating CMR with firing rate, that the latter should decline with brain size with an exponent around 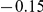 [19]. This implies that neurons in larger brain are on average less active than neurons in smaller brains. Such sparse neural representations may be advantageous in terms of saving the metabolic energy [18], [60], [61]. At the same time, what may be related, neural activity is distributed in such a way that both the average energy per neuron and the average blood flow per neuron are approximately invariant with respect to brain size (Fig. 5; Table 3, [26]). Additionally, average firing rate should be inversely proportional to the average blood transit time 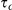 through a capillary, because both of them scale reversely with brain size (Table 3). Thus, it appears that global timing in neural activities should be correlated with the timing of cerebral blood flow. These general considerations suggest that apart from structural neuro-vascular coupling there is probably also a significant dynamic coupling. This conclusion is qualitatively compatible with experimental observations in which enhanced neural activity is invariably accompanied by increase in local blood flow [62].

### 4. Relationship to brain functional imaging

The interdependencies between brain metabolism, blood flow, and capillary parameters can have practical meaning. Currently existing techniques for non-invasive visualization of brain function, such as PET or fMRI, are associated with measurements of blood flow CBF and oxygen consumption CMR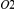. It turns out that during stimulation of a specific brain region, CBF increases often, but not always, far more than CMR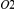 [63]. However, both of them increase only by a small fraction in relation to the background activity, even for massive stimulation [62], [63]. This phenomenon was initially interpreted as an uncoupling between blood perfusion and oxidative metabolism [64]. Later, it was shown that this asymmetry between CBF and CMR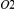 can be explained in terms of mechanistic limitations on oxygen delivery to brain tissue through blood flow [65]. We can provide a related, but simpler explanation of these observations that involves physical limitations on the relative changes in capillary oxygen pressure and radius.

During brain stimulation, both CBF and CMR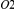 change by 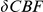 and 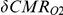, which are according to Eqs. (1) and (8) related to modifications in capillary radius (from 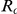 to 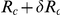), and changes in partial oxygen pressure (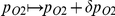). The density of perfused capillary length 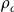 remains constant for normal neurophysiological conditions. Accordingly, a small fraction of blood flow change is
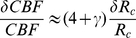
(9)and similarly, a small fractional change in the oxygen metabolic rate is:
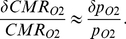
(10)


In general, oxygen pressure increases with increasing capillary radius, in response to increase in blood flow CBF. This relationship can have a complicated character. We simply assume that 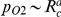, where the unknown exponent 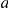 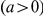 contains all the non-linear effects, however complicated they are. Thus, a small fractional change in oxygen pressure can be written as 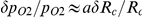. As a result, we obtain
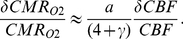
(11)


If partial oxygen pressure 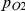 depends on capillary radius linearly or sublinearly, i.e., if 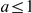, then the fractional increase in oxygen metabolism is significantly smaller than a corresponding increase in cerebral blood flow. This case corresponds to the experimental reports showing that this ratio is 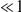, for example, in the visual cortex (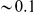) [66] and in the sensory cortex (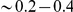) [64], [67]. If, in turn, 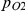 depends on 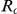 superlinearly, i.e. if 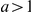, then the coefficient 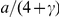 in Eq. (10) can be of the order of unity. Such cases have been also reported experimentally during cognitive activities [42] or anesthesia [68], [69].

## Materials and Methods

The ethics statement does not apply to this study. CBF data were collected from different sources: for mouse [70], rat [71], rabbit [72], cynomolgus monkey [73], rhesus monkey [74], pig [75], and human [76]. Cerebral capillary characteristics were obtained from several sources: for mouse [14], [51], rat [15], [77], [32], cat [40], [78], dog [79], rhesus monkey [80], and human [33], [34]. Data for calculating neuron densities were taken from [24], [25], [41], [52], [81], [82]. Cortical volume data (for 2 hemispheres) are taken from [52], [41], [81]. Their values are: mouse 0.12 cm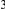, rat 0.42 cm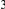, rabbit 4.0 cm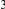, cat 14.0 cm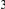, cynomolgus monkey 21.0 cm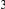, dog 35.0 cm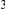, rhesus monkey 42.9 cm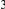, pig 45.0 cm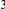, human 571.8 cm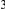. All the numerical data are provided in the Supporting Information (Tables S1, S2, and S3).

## Supporting Information

Appendix S1 (TEX)

Table S1 Regional cerebral blood flow CBF in mammals.
(TEX)

Table S2 Cerebral capillary and neural characteristics in mammals.
(TEX)

Table S3 Arterial partial oxygen pressure and average cortical CBF per neuron.
(TEX)
